# Nicotinamide Mononucleotide Combined With *Lactobacillus fermentum* TKSN041 Reduces the Photoaging Damage in Murine Skin by Activating AMPK Signaling Pathway

**DOI:** 10.3389/fphar.2021.643089

**Published:** 2021-03-25

**Authors:** Xianrong Zhou, Hang-Hang Du, Luyao Ni, Jie Ran, Jian Hu, Jianjun Yu, Xin Zhao

**Affiliations:** ^1^ Chongqing Collaborative Innovation Center for Functional Food, Chongqing University of Education, Chongqing, China; ^2^ Chongqing Engineering Research Center of Functional Food, Chongqing University of Education, Chongqing, China; ^3^ Chongqing Engineering Laboratory for Research and Development of Functional Food, Chongqing University of Education, Chongqing, China; ^4^ Department of Plastic Surgery, Chongqing Huamei Plastic Surgery Hospital, Chongqing, China; ^5^ Effepharm (Shanghai) Co., Ltd., Shanghai, China

**Keywords:** nicotinamide mononucleotide, *lactobacillus* fermentum, in vitro antioxidant, UVB, AMP, skin

## Abstract

Long-term exposure to UVB (280–320 nm) can cause oxidative skin damage, inflammatory injury, and skin cancer. Research on nicotinamide mononucleotide (NMN) and lactic acid bacteria (LAB) with regard to antioxidation, anti-inflammation, and prevention of other age-related diseases has received increasing attention. In the present study, the *in vitro* antioxidant analysis showed that NMN combined with *Lactobacillus fermentum* TKSN041 (*L. fermentum* TKSN041) has a high scavenging ability on hydroxyl (OH), 2, 2′-azino-bis (3-ethylbenzthiazoline-6-sulphonic acid) diammonium salt (ABTS) and 1, 1-diphenyl-2-picrylhydrazyl (DPPH), and it also possess a good total antioxidant capacity. The animal experimental results show that NMN combined with LAB maintained normal liver morphology of mice and reduced pathological damage to murine skin. NMN combined with LAB significantly increased the serum levels of total superoxide dismutase (T-SOD), catalase (CAT), and interleukin (IL)-10, but reduced the levels of malondialdehyde, advanced glycation end products, tumor necrosis factor (TNF)-α, and IL-6. NMN combined with LAB increased T-SOD, CAT, IL-10, Na^+^-K^+^-ATPase, and NAD^+^ levels in the skin, but reduced TNF-α level in the skin. NMN combined with LAB increased the mRNA expression levels of SOD1, CAT, glutathione (GSH), inhibitor of NF-κB (IκB-α), IL-10, AMP-activated protein kinase (AMPK), adaptor protein, phosphotyros ineinteraction, PH domain and leucine zipper containing 1 (APPL1), peroxisome proliferator-activated receptor γ co-activator-1α (PGC-1α), and forkhead transcription factor O (FOXO) in the skin and liver, but decreased the mRNA expression levels of nuclear factor (NF)-κBp65, TNF-α, IL-6, and rapamycin target protein (mTOR). NMN combined with LAB increased the protein expression levels of AMPK, IκB-α, SOD1, and CAT in the skin tissues and reduced protein expression of NF-κBp65. NMN combined with *L. fermentum* TKSN041 improved murine skin damage caused by UVB irradiation, and the protective mechanism may be related to activation of the AMPK signaling pathway. The results of this study are expected to provide a reference for preventing and the treating skin photoaging.

## Introduction

The skin is one of the largest and most complex organs in the human body, accounting for approximately 15% of body weight. It is also the first line of defense against environmental damage (Vollmer et al., 2018). Skin aging is an important part of body aging, which is not only detrimental to beauty but also closely related to the occurrence of many skin diseases, such as seborrheic keratosis, solar keratosis, basal cell carcinoma (BCC), and squamous cell carcinoma (SCC) (Gossai et al., 2016). Photoaging refers to premature aging of the skin due to repeated light exposure. Its clinical manifestations, histopathology, and biochemical changes are different from the natural aging of skin. Photoaging reduces the amount of mature type I collagen and elastic fibers in the dermis of the skin. The clinical characteristics of photoaging occur mainly on exposed skin, such as the face, neck, and forearm, where rough skin, loss of elasticity, deepening and thickening of wrinkles, a leather-like appearance, pigmentation, and dilated capillaries can occur (Qin et al., 2018). Studies have shown that approximately 65% of patients with melanoma and 90% of patients with non-melanoma skin cancers, including BCC and SCC, are associated with skin photoaging (Damiani and Ullrich, 2016). Many external factors are attributed to photoaging of the skin, such as ultraviolet (UV), infrared, chemical smog, dust, and smog, among which UV radiation is the most significant (Markiewicz and Idowu, 2018).

Anti-aging skin has become a research hotspot of many scholars and clinicians, and it has also captivated the attention of many beauty seekers. Therefore, establishing a practical photoaging model is particularly important to investigate the occurrence, development mechanism, and screening of anti-photoaging agents. The occurrence and development of skin photoaging mediated by UV radiation involves multiple pathways, including apoptosis, proliferation, autophagy, DNA repair, checkpoint signal transduction, cell transduction, and inflammation. UV radiation is generally categorized according to wavelength into long-wave UVA (315–400 nm), medium-wave UVB (280–315 nm), and short-wave UVC (200–280 nm). Although UVB radiation (280–315 nm) accounts for only 1–2% of the UV rays of the Sun, it is considered to be the main environmental carcinogen that causes skin cancer and is related to the occurrence and the development of tumors (Panich et al., 2016; Gherardini et al., 2019). Patients with chronic immunosuppression who live in areas with intense sunshine are more likely to develop skin redness and swelling. The incidence of skin cancer is high among organ transplant recipients receiving continuous immunosuppressive therapy (Surdu et al., 2013). Experimental models are the most widely used photoaging models. This type of experimental model often uses UVB because the skin tissue changes caused by UVB are very similar to photoaging of human skin (Kim et al., 2019b).

Nicotinamide ribonucleotide (NMN) is synthesized by nicotinamide and 5′-phosphate pyrophosphate through nicotinamide phosphotransferase and is a key intermediate of NAD^+^. NMN enhances NAD^+^ biosynthesis and improves various pathologies in murine disease models, such as myocardial and cerebral ischemia, Alzheimer’s disease, other neurodegenerative diseases, and diabetes (Braidy et al., 2019). Most of the pharmacological effects of NMN are carried out by promoting the synthesis of NAD^+^ because the direct administration of high doses of NAD^+^ causes side-effects, such as insomnia, fatigue, and anxiety, and the penetrating ability of NAD^+^ into the plasma membrane is poorer than that of NMN (Mills et al., 2016). The newly discovered anti-aging and longevity properties in murine models make NMN more attractive. Studies have shown that supplementing with NMN improves the metabolic and stress responses of mice with age; thus, NMN is considered to be a promising method for treating age-related physiological dysfunction and diseases (Tarantini et al., 2019).

Lactic acid bacteria (LAB) are Gram-positive bacteria that are widely distributed in nature. They have important application value in fields closely related to humans, such as industry, agriculture, animal husbandry, food, and medicine. LAB have many goods effects on body health, such as balance of the intestinal microbiota, regulation of immune system, reducing the risk of tumors, and lowering of serum cholesterol (Thomas et al., 2017). Otherwise, anti-aging and antioxidant activity both are important probiotic functions of LAB, which have attracted the attention of researchers (Azat et al., 2016). Studies have reported that live bacteria or heat-killed bacteria can improve the skin condition of Japanese women. Murine experiments have also shown that this strain reduces the incidence of skin ulcers as well as reduces osteoporosis and hair loss (Kimoto-Nira et al., 2012). In another study, a strain named *Lactobacillus plantarum* MA2 has high antioxidant potential (Slattery et al., 2019). The serarches for new LAB and studies on new LAB with human health functions have also attracted increasing attention in the fields of food and medicine.

Previous studies have shown that intestinal microbiota can affect skin health (Hayashi et al., 2017). A recently published study found that the application of TLR7 agonists to the skin of mice induces psoriatic dermatitis, which can affect the composition of intestinal immune cells and microbiota, leading to subsequent DSS-induced colitis. These findings show a link between gut microbiota modulation and skin inflammation (Kiyohara et al., 2018). At the same time, ultraviolet radiation will also lead to changes in the diversity and abundance of intestinal microbiota, which may lead to skin disease (Jung et al., 2017). On the other hand, changes in the intestinal microbiota and intake of probiotics can affect the immune response of the skin. In fact, lipoteichoic acid (LTA) from LGG (*Lactobacillus rhamnosus*) can prevent skin tumors in mice exposed to long-term ultraviolet rays (Friedrich et al., 2019).

Vitamin C, also called ascorbic acid, is a water-soluble electron donor (Lykkesfeldt et al., 2014). Acting as the role of a reductant is the most obvious biological function known to vitamin C (Lane and Richardson, 2014). Ascorbic acid is an effective antioxidant, it can remove many harmful free radicals and active oxygen in the body (Moser and Chun, 2016). In addition, vitamin C can promote the production of alpha-tocopherol (vitamin E), thereby inhibiting lipid peroxidation. Therefore, in many antioxidant experiments, vitamin C is selected as the positive control (Sealey and Gatlin, 2002; Bleilevens et al., 2019).

In this study, a murine model of skin damage induced by UVB was used. The mice were given an intragastric treatment of the nicotinamide single nucleotide combined with a *L. fermentum* TKSN041 bacterial suspension. We used serum and skin oxidation indicators, inflammation indicators, and skin and liver mRNA expression levels and protein expression of related genes to evaluate the effect of NMN combined with *L. fermentum* TKSN041 on UVB-induced skin damage in mice. We aim to identify new methods to prevent skin aging or develop a new micro-ecology of NMN combined with LAB preparations to provide a theoretical reference and available raw materials.

## Materials and Methods

### Experimental Strain

L. fermentum TKSN041 was isolated and purified from Xinjiang’s naturally fermented yak yoghurt, and was stored in the China General Microbial Member Center (CGMCC, Beijing, China) (deposit number 18222).

### NMN Source

NMN was provided by EffePharm Co., Ltd. (Shanghai, China), and the purity of the NMN was determined by high-performance liquid chromatography (purity >98.5%).

### Preparation of Strain Intracellular Extract

With a slight modification according to the literature method (Xu et al., 2018; Barache et al., 2020), the bacteria were collected by centrifugation and washed three times with PBS (pH 7.4, 0.01M) after *L. fermentum* TKSN041 was cultured three times. Then the concentration of the bacterial suspension was adjusted to 1 × 10^9^ CFU/ml, and it was treated in an ultrasonic disintegrator (KunShan Ultrasonic Instruments Co., Ltd., China) at 100 W for 30 min. At last, the bacterial suspension was centrifuged at 4°C, 10,000 rpm for 30 min, and the supernatant was collected (intracellular extract of 10^9^ cells).

### *In Vitro* Antioxidant Analysis

According to the previous works (Manafikhi et al., 2017; Liu et al., 2019), the 1,1-diphenyl-2-picrylhydrazyl (DPPH) free-radical scavening activity, •OH free-radical scavenging activity, 2,2′-azino-bis(3-ethylbenzothiazoline-6-sulfonic acid (ABTS) free-radical scavenging activity, and total antioxidant capacity of NMN solution (30 mg/ml), *L. fermentum* TKSN041 intracellular extract, and NMN soulution combined with *L. fermentum* TKSN041 intracellular extract (1:1) were both determined. The absorbance of the solution was determined by a multi-function micro-plate reader (Thermo Fisher Scientific, New York, United States). Ascorbic acid (0.2 mg/ml) was used as positive control. The measurements were performed three times and averaged.

### Experimental Animals

Sixty female 7-week-old Kunming mice were purchased from the Experimental Animal Center of Chongqing Medical University [Chongqing, China, SCXK (YU) 2018-0003]. The mice were kept under constant temperature and humidity conditions (temperature 25 ± 2°C, relative humidity 50 ± 5%) and a 12 h light/dark cycle. They were allowed to eat standard rat chow and drink water freely.

### UVB-Induced Skin Damage

**FIGURE 1:**
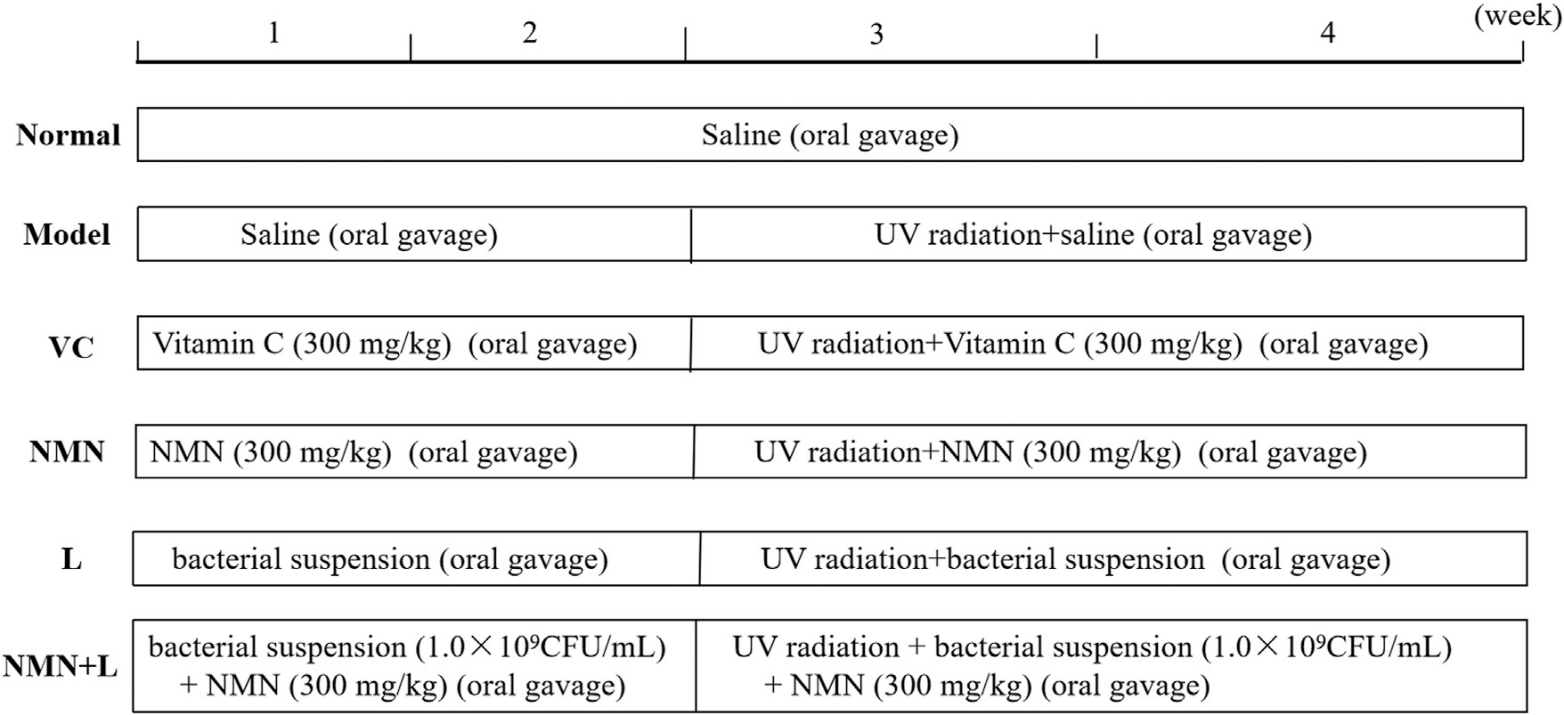
Experimental test treatments. VC: mice treated with vitamin C (300 mg/kg); NMN: mice treated with nicotinamide mononucleotide (300 mg/kg); L: mice treated with *L. fermentum* TKSN041 (1.0 × 109 CFU/ml); NMN + L: mice treated with nicotinamide mononucleotide (300 mg/kg) and *L. fermentum* TKSN041 (1.0 × 109 CFU/ml).

Sixty mice were randomly divided into six groups, with 10 mice in each group, including the normal group, the model group, the vitamin C (VC) group, the NMN group, the *L. fermentum TKSN041* group (L), and the NMN + *L. fermentum* TKSN041 group (NMN + L). The UVB modeling method was as follows (Figure 1). The entire experimental period was 4 weeks. UV radiation equipment was used to establish the skin damage model in the other groups of mice except the normal group beginning on week 3, and irradiation (120 mJ/cm^2^/h) was performed for 2 h every day (Kawashima et al., 2018). Before UV irradiation, an electric shaver was used to shave about 2 cm^2^ of hair from the back of the mouse. The specific treatment methods for each group of mice were as follows: the normal group and the model group drank water and ate the diet; mice in the VC group were given the VC solution at a dose of 30 mg/ml per day; mice in the NMN group were given the NMN solution at a dose of 30 mg/ml per day; mice in the L group were orally dosed with 1.0 × 10^9^ CFU/ml bacterial; mice in NMN + L group were orally dosed with NMN at the same dose, and the mice were orally gavaged with 1.0 × 10^9^ CFU/ml bacterial suspension every day. In addition, all mice received gavage treatment and then received ultraviolet radiation 1 h later. All experiments were approved by the Chongqing Functional Food Collaborative Innovation Center.

### Sample Collection

After 4 weeks, the mice were sacrificed by removing the spine. Whole blood samples were collected through the inferior vena cava, and serum samples were collected by centrifugation and stored at –80°C until use. We dissected the mouse and removed the liver and the hairless back skin. We cut the soybean-sized liver and skin tissues and soaked them in 4% paraformaldehyde solution; the remaining liver and skin tissues were stored at –80°C for future use.

### Histological Tissue Sectioning

The liver was stained with hematoxylin and eosin (H&E), and the skin was stained with H&E, Masson’s trichrome, and toluidine blue. We used a microscope to observe the tissue pathology. Finally, the histology scores were evaluated by Professor Qian Yu.

### Determination of Serum Indicators

The levels of total-superoxide dismutase (T-SOD), catalase (CAT), malondialdehyde (MDA), and advanced glycation end products (AGEs) were measured in murine serum according to the manufacturer’s instructions (NanJing JianCheng Bioengineering Institute, NanJing, China). The levels of tumor necrosis factor (TNF)-α, interleukin (IL)-6, and IL-10 in murine serum were determined according to the enzyme-linked immunosorbent assay (ELISA) kit instructions (Shanghai Enzyme-Linked Biotechnology Co., Ltd., Shanghai, China).

### Determination of Related Indicators in Murine Skin

The levels of T-SOD, CAT, Na^+^-K^+^-ATPase, and NAD^+^ were determined in murine skin according to the manufacturer’s instructions (NanJing JianCheng Bioengineering Institute, China). The levels of TNF-α and IL-10 in the skin tissue were determined according to the ELISA kit instructions (Shanghai Enzyme-Linked Biotechnology Co., Ltd., China).

### Quantitative Reverse Transcription-Polymerase Chain Reaction Analysis

The liver and skin tissues were homogenized, and total RNA was extracted with Trizol reagent. RNA was reverse transcribed into cDNA using a cDNA kit (Thermo Fisher Scientific, Inc., Waltham, MA, United States). Then, we mixed 1 μl cDNA, 10 μl TaqMan™ Multiplex Master Mix, 2 µl of 10 µM primer, and 7 µl ddH_2_O, and performed amplification on a real-time fluorescence quantitative PCR instrument (Thermo Fisher Scientific). The amplification conditions were: 95°C deformation for 15°s, 55°C annealing for 30°s, 72°C extension for 35°s, for a total of 40 cycles. Finally, the relative expression of each target gene was calculated by the 2^−ΔΔCT^ method with β-actin as the internal reference gene (Li et al., 2011). The primer sequences used in this experiment are shown in Table 1.

**TABLE 1:** Sequences of the primers used for the mice liver and skin tissues.

Gene	Sequences
*NF-κBp65*	F: 5´- GAG​GCA​CGA​GGC​TCC​TTT​TCT -3´
	R: 5´- GTA​GCT​GCA​TGG​AGA​CTC​GAA​CA -3´
*IκB-α*	F: 5´-TGA​AGG​ACG​AGG​AGT​ACG​AGC-3´
	R: 5´-TGC​AGG​AAC​GAG​TCT​CCG​T-3´
*TNF-α*	F: 5´-CAG​GCG​GTG​CCT​ATG​TCT​C-3´
	R: 5´-GCT​GCA​ACA​GGG​GGT​AAC​AT-3´
*IL-6*	F: 5´-CTG​CAA​GAG​ACT​TCC​ATC​CAG-3´
	R: 5´-AGT​GGT​ATA​GAC​AGG​TCT​GTT​GG-3´
*IL-10*	F: 5´-CTT​ACT​GAC​TGG​CAT​GAG​GAT​CA-3´
	R: 5´-GCA​GCT​CTA​GGA​GCA​TGT​GG-3´
*SOD1*	F: 5´-AAC​CAG​TTG​TGT​TGT​CAG​GAC-3´
	R: 5´-CCA​CCA​TGT​TTC​TTA​GAG​TGA​GG-3´
*CAT*	F: 5´-GGA​GGC​GGG​AAC​CCA​ATA​G-3´
	R: 5´-GTG​TGC​CAT​CTC​GTC​AGT​GAA-3´
*GSH*	F: 5´-CCA​CCG​TGT​ATG​CCT​TCT​CC-3´
	R: 5´-AGA​GAG​ACG​CGA​CAT​TCT​CAA​T-3´
*AMPK*	F: 5´-GTC​AAA​GCC​GAC​CCA​ATG​ATA-3´
	R: 5´-CGT​ACA​CGC​AAA​TAA​TAG​GGG​TT-3´
*mTOR*	F: 5´-CAG​TTC​GCC​AGT​GGA​CTG​AAG-3´
	R: 5´-GCT​GGT​CAT​AGA​AGC​GAG​TAG​AC-3´
*PGC-1α*	F: 5´-TAT​GGA​GTG​ACA​TAG​AGT​GTG​CT-3´
	R: 5´-GTC​GCT​ACA​CCA​CTT​CAA​TCC-3´
*APPL1*	F: 5´-AGC​CAG​TGA​CCC​TTT​ATA​TCT​GC-3´
	R: 5´-AGG​TAT​CCA​GCC​TTT​CGG​GTT-3´
*FOXO*	F: 5´-CCC​AGG​CCG​GAG​TTT​AAC​C-3´
	R: 5´-GTT​GCT​CAT​AAA​GTC​GGT​GCT-3´
*β-actin*	F: 5´-CAT​GTA​CGT​TGC​TAT​CCA​GGC-3´
	R: 5´-CTC​CTT​AAT​GTC​ACG​CAC​GAT-3´

### Western Blot

After extracting the total protein from colon tissue with RIPA tissue lysate, the protein concentration was determined using the BCA protein quantification kit (Beijing Solarbio Science and Technology Co., Ltd., Beijing, China). A sodium dodecyl sulfate-polyacrylamide gel electrophoresis gel kit (Thermo Fisher Scientific, Inc., Waltham, MA, United States) was used to prepare the gel, and a 50 μg protein solution was combined with the gel for electrophoresis. The proteins on the gel were transferred to a PVDF membrane (Millipore, Billerica, MA, United States). Then, 5% skim milk was used to seal the protein-containing PVDF membrane for 1 h (25°C, 75 r/min). The primary antibody was incubated with the membrane for 2 h (37°C, 75 r/min), and the secondary antibody was incubated for 1 h (30°C, 80 r/min). Finally, the color developing solution was prepared according to the manufacturer’s instructions (Beijing Solarbio Science and Technology Co., Ltd., Beijing, China), and imaging and shooting were performed using the Tiangen chemiluminescence imaging system (Tanon Science and Technology Co., Ltd., Shanghai, China). The band image grayscale analysis was performed using ImageJ software (National Institutes of Health, Bethesda, MD, United States), and β-actin was used as the internal reference protein to calculate the relative expression of the target protein (Simiczyjew et al., 2014).

### Statistical Analysis

The SPSS17.0 (IBM Corp., Armonk, NY, United States) statistical software was used to analyze the related oxidative stress indicators and inflammation indicators in the serum and skin of aging mice. The comparison between multiple groups was performed by analysis of variance. Duncan test was used for multiple comparisons. The remaining data were calculated and analyzed using Pad Prism 7.0 (Graph Pad Software, La Jolla, CA, United States) software, group differences were also analyzed by one-way analysis of variance (ANOVA) followed by Duncan’s multiple comparison test, and “Compare the mean of each column with the mean of a control column” was chosen as the “followup tests.” All of the values are expressed as mean ± standard deviation (x̅ ± SD), and a *p*-value <0.05 was considered significant.

## Results

### Anti-Oxidant Activities

**FIGURE 2:**
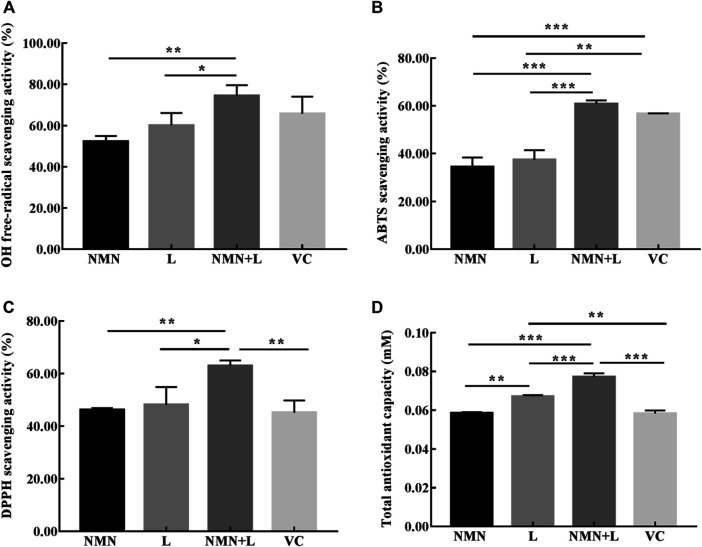
*In vitro* antioxidant activities of each experimental group. **(A)** hydroxyl (OH) radical; **(B)** 2, 2′-azino-bis (3-ethylbenzthiazoline-6-sulphonic acid) diammonium salt (ABTS) radical; **(C)** 1, 1-diphenyl-2-picrylhydrazyl (DPPH) radical; **(D)** total antioxidant capacity. The data were calculated and analyzed using Pad Prism 7.0 (Graph Pad Software, La Jolla, CA, United States) software, group differences were also analyzed by one-way analysis of variance (ANOVA) followed by Duncan’s multiple comparison test. Values are expressed as mean ± standard deviation (*N* = 3/group). *p < 0.05; **p < 0.01; ***p < 0.001. NMN: nicotinamide mononucleotide solution (30 mg/ml); L: *L. fermentum TKSN041* intracellular extract of 10^9^ cells; VC: ascorbic acid (0.2 mg/ml); NMN + L: nicotinamide mononucleotide soulution (30 mg/ml) combined with *L. fermentum TKSN041* intracellular extract of 10^9^ cells.

OH, ABTS, and DPPH are usually used to test the free radical scavenging ability of bioactive substances, which are also important indexes to measure antioxidant activity (Pu et al., 2019). As shown in Figures 2A–D, the OH, ABTS, DPPH free-radical scavenging activities, and total antioxidant capacity were varied between different samples, in which NMN + L were both higher than that of NMN or L alone. The results indicate that NMN solution combined with L. fermentum TKSN041 intracellular extract can synergistically enhance the ability to clear free radicals and show good *in vitro* antioxidant effects.

### Murine Liver Organ Index and Pathological Morphology

**FIGURE 3:**
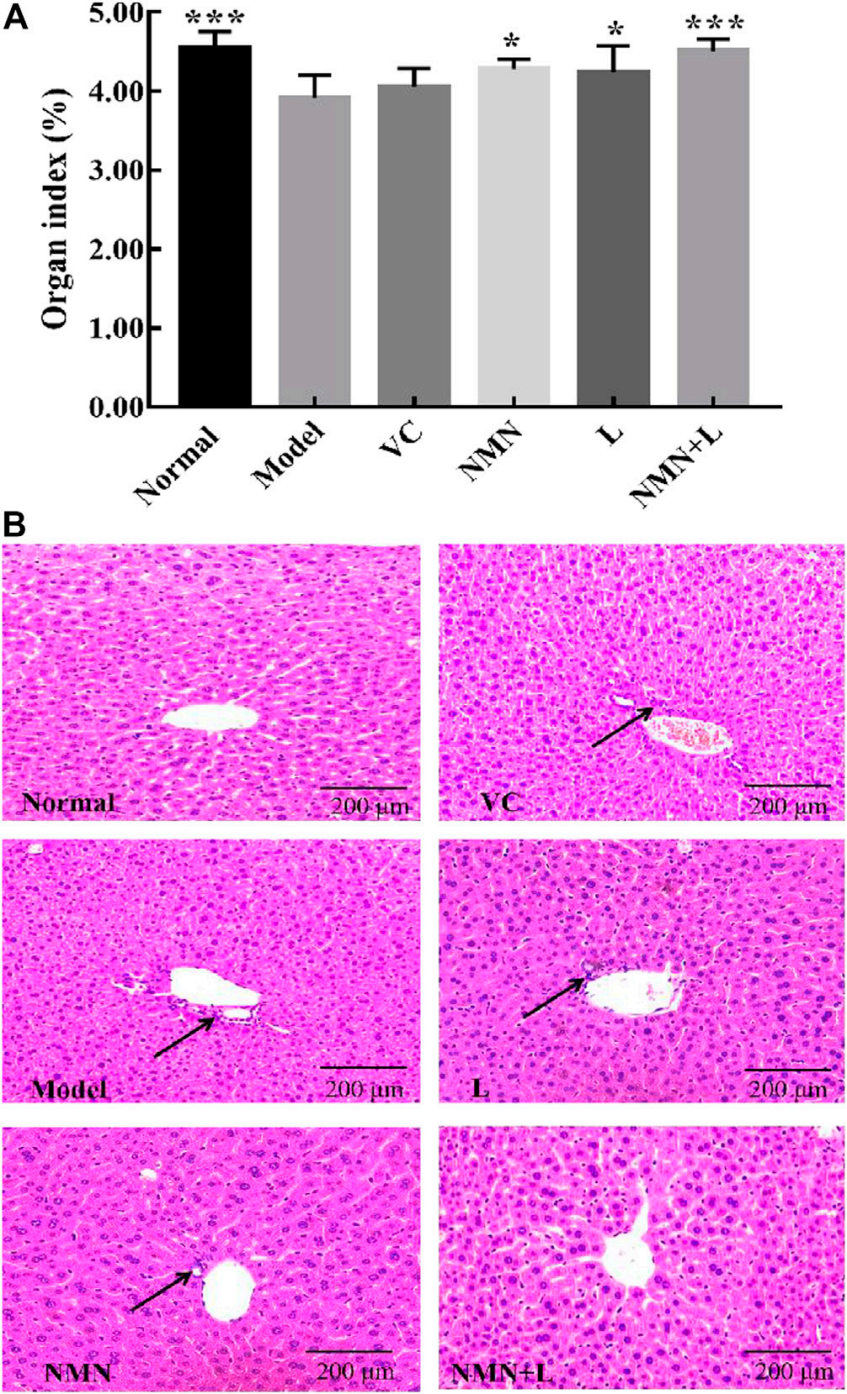
Organ index and pathological morphology of the mice liver. Magnification ×100. **(A)** Liver organ indices. **(B)** Liver pathological morphology. The data were calculated and analyzed using Pad Prism 7.0 (Graph Pad Software, La Jolla, CA, United States) software, group differences were also analyzed by one-way analysis of variance (ANOVA) followed by Duncan’s multiple comparison test. *p < 0.05 compared to the model group; ***p < 0.001 compared to the model group. VC: mice treated with vitamin C (300 mg/kg); NMN: mice treated with nicotinamide mononucleotide (300 mg/kg); L: mice treated with *L. fermentum TKSN041* (1.0 × 10^9^ CFU/ml); NMN + L: mice treated with nicotinamide mononucleotide (300 mg/kg) and *L. fermentum* TKSN041 (1.0 × 10^9^ CFU/ml).

Animal organ weight and organ index are important basic indicators for biomedical research. Aging usually causes cell degeneration, atrophy, number reduction, tissue dehydration, and ultimately leads to the weight loss of most organs (López-Lluch and Navas, 2016). Figure 3A shows that the liver organ index scores of mice in the normal group were significantly higher than those in the model group. The liver organ index scores of the VC group, the NMN group, the L group, and the NMN + L group increased to varying degrees compared with the model group, of which the NMN + L group scores were significantly elevated, and no significant difference was observed in the normal group.

Studies have shown that accelerated human aging process usually promotes hepatic dysfunction and makes hepatic cells present a pro-inflammatory state, and pathological observation can directly reflect the apparent abnormality of the liver (Maeso-Díaz et al., 2018). As shown in Figure 3B, the liver of the normal group of mice was intact. The liver cells were arranged neatly and orderly around the central vein in a satellite emission pattern. The nuclei were large and round, and there was no infiltration of inflammatory cells. The liver cells of mice in the model group were disordered. The liver cells around the central vein were partially necrotic and infiltrated by inflammatory cells. The overall structural integrity of the liver was worse than that in the normal group. The liver cell structure of mice in the VC group, the NMN group, and the L group was better than that in the model group, but some cell necrosis and inflammatory cell infiltration were present. The liver morphology of the mice in the NMN + L group was significantly more complete than that in the model, the NMN, the L and the VC groups, and there were almost no cell necrosis or inflammatory cell infiltration. The overall liver structure was similar to that in the normal group.

### Skin Pathological Morphology

Ultraviolet radiation is considered to be the most harmful factor that induces cellular senescence and skin aging. The anile skin usually shows shrinkage and lysis of collagen fibers and increase of mast cells. At present, H&E, TB, and Masson’s staining are commonly used methods to observe the pathology of the skin (Li et al., 2018). Therefore, these three methods were used to evaluate the degree of damage to the skin of mice by ultraviolet rays, and to explore the improvement effect of *L. fermentum* TKSN041 combined with NMN on the damaged skin.

**FIGURE 4:**
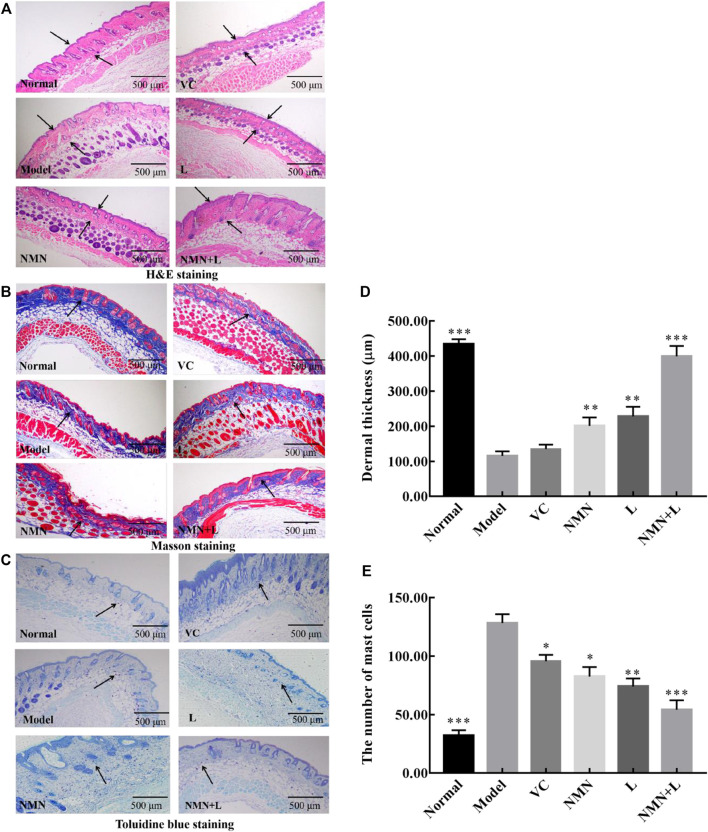
Skin pathological morphology. Magnification ×40. **(A)** : H&E staining of the skin;. **(B)**: Masson’s staining of the skin; **(C)**: toluidine blue staining; **(D)**: Dermal thickness of the skin; **(E)** The number of mast cells. The data were calculated and analyzed using Pad Prism 7.0 (Graph Pad Software, La Jolla, CA, United States) software, group differences were also analyzed by one-way analysis of variance (ANOVA) followed by Duncan’s multiple comparison test. *p < 0.05 compared to the model group; **p < 0.01 compared to model group; ***p < 0.001 compared to the model group. VC: mice treated with vitamin C (300 mg/kg); NMN: mice treated with nicotinamide mononucleotide (300 mg/kg); L: mice treated with *L. fermentum TKSN041* (1.0 × 10^9^ CFU/ml); NMN + L: mice treated with nicotinamide mononucleotide (300 mg/kg) and *L. fermentum TKSN041* (1.0 × 10^9^ CFU/ml).

Figures 4A,D show that the normal group of mice had a complete skin structure, a thin epidermal layer, and no excessively keratinized stratum corneum; there was also a thicker dermal layer with a complete collagen bundle morphological structure, an orderly arrangement, and an even distribution. The thickness of the dermal layer was significantly thinned in the model group. The number of collagen fiber bundles decreased significantly, the arrangement of subcutaneous tissue was disordered, and the boundary was not obvious. In addition, infiltration of inflammatory cells was seen around the appendages. The thickness of the skin dermis in the VC group, NMN group, and L group increased compared with that in the model group, but the collagen fibers were loosely dispersed. The thickness of the skin dermis layer in the NMN + L group increased significantly compared with the model, NMN, L, and VC groups; no fracture, shrinkage, or adhesion of the collagen fiber bundles was observed and the overall structure approached that of the normal group.

The collagen fibers stained blue-violet after Masson’s staining. Figure 4B shows that the normal group had a large number of collagen fibers evenly and orderly distributed. Significantly fewer collagen fibers were observed in the model group than that in the normal group, and breakage and shrinkage occurred. Significantly fewer collagen fibers were observed in the skin dermis of the VC group, NMN group and L group than that in the normal group, but more were seen than in the model group. The number of collagen fibers in the skin dermal layer of the NMN + L group increased more than that in the model, NMN, L, and VC groups. The arrangement was more orderly, and there was almost no shrinkage or fracture.

According to the TB staining results (Figures 4C,E), the number of mast cells in the dermis of the model group increased significantly compared with the normal group, indicating that UVB irradiation induced production of skin mast cells, which leads to skin inflammation. The number of skin mast cells in the mice of the VC group, NMN group, and L group decreased compared with that in the model group, but the number was significantly higher than that in the normal group. After the mice were treated with NMN and LAB, the number of mast cells in the skin of the mice in the NMN and NMN + L groups decreased significantly, and the results were similar to those of the normal group.

### Serum Levels of Oxidative Stress and Inflammation Indicators

In order to determine whether *L. fermentum* TKSN041 combined with NMN treatment can cause changes in oxidative stress indicators and inflammatory cytokine release in UVB-induced skin inflammation, the serum levels of T-SOD, CAT, MDA, AGEs, TNF-α, IL-6, and IL-10 of mice in the normal group, model group, NMN group and NMN + L group were measured. Table 2 shows that serum levels of T-SOD, CAT, and IL-10 in the model group were significantly lower than those in the normal group, while the levels of MDA, AGEs, TNF-α, and IL-6 were significantly higher than those in the normal group (*p* < 0.05). Serum T-SOD, CAT, and IL-10 levels of mice in the VC, NMN, L and NMN + L groups increased compared with the model group, whereas the levels of MDA, AGEs, TNF-α, and IL-6 decreased. Notably, serum T-SOD, CAT, IL-10, MDA, AGEs, TNF-α, and IL-6 levels of mice in the NMN + L group were closer to those in the normal group, in which T-SOD activity was significantly higher than that in the normal group (*p* < 0.05).

**TABLE 2:** Serum levels of the oxidative stress and inflammatory indices in mice.

Group	T-SOD (U/mL)	CAT (U/mL)	MDA (mmol/L)	AGEs (pg/ml)	TNF-α (ng/L)	IL-6 (pg/ml)	IL-10 (pg/ml)
Normal	93.72 ± 16.79^b^	39.68 ± 5.20^a^	11.38 ± 2.55^b^	35.40 ± 7.15^b^	238.87 ± 67.66^c^	17.36 ± 5.53^c^	320.38 ± 47.29^a^
Model	50.59 ± 8.49^c^	19.49 ± 4.65^c^	30.48 ± 7.83^a^	126.93 ± 12.80^a^	534.70 ± 86.93^a^	73.72 ± 9.09^a^	136.85 ± 27.68^c^
VC	86.65 ± 15.80^b^	25.19 ± 4.31^b^	25.90 ± 8.19^a^	116.81 ± 14.22^a^	309.10 ± 59.64^b^	50.12 ± 8.02^b^	141.07 ± 16.49^c^
NMN	173.31 ± 13.56^a^	33.09 ± 5.30^b^	15.80 ± 3.26^cd^	32.83 ± 7.38^b^	253.23 ± 44.90^bc^	19.21 ± 5.14^c^	163.20 ± 40.08^bc^
L	140.75 ± 13.01^b^	35.90 ± 5.47^ab^	21.61 ± 3.87^bc^	37.16 ± 7.21^b^	249.26 ± 39.85^bc^	18.84 ± 5.07^c^	170.25 ± 34.98^bc^
NMN + L	186.04 ± 29.54^a^	36.31 ± 4.51^a^	13.33 ± 3.31^b^	19.52 ± 3.25^c^	216.96 ± 31.50^c^	13.09 ± 3.94^c^	187.30 ± 27.74^b^

Values are mean ± standard deviation (N = 10/group). The difference in variance between the two groups was significant (*p* < 0.05).

^a-c^Mean values with different letters in the same column are significantly different (*p* < 0.05) according to Duncan’s honestly significantly different test. VC: mice treated with vitamin C (300 mg/kg).

NMN, mice treated with nicotinamide mononucleotide (300 mg/kg); L, mice treated with L. fermentum TKSN041 (1.0 × 10^9^ CFU/ml); NMN + L, mice treated with nicotinamide mononucleotide (300 mg/kg) and L. fermentum TKSN041 (1.0 × 10^9^ CFU/ml); T-SOD, total superoxide dismutase; CAT, catalase; MDA, malondialdehyde; AGEs, advanced glycation end products; TNF-α, tumor necrosis factor α; IL-6, interleukin 6; IL-10, interleukin 10.

### Skin Levels of Related Oxidative Stress Indicators and Inflammation Indicators

Long-term UVB radiation can induce severe oxidative stress and inflammatory symptoms in the skin. Therefore, the evaluation of these two indicators in the skin tissue can reflect the specific effects of *L. fermentum* TKSN041 combined with NMN on UVB-induced skin damage. As shown in Table 3, the T-SOD, CAT, IL-10, Na^+^-K^+^-ATPase, and NAD^+^ levels in the skin tissue of the normal group of mice were 26.68 ± 6.52 U/mgprot, 23.07 ± 3.41 U/mgprot, 632.98 ± 82.99 pg/ml, 0.86 ± 0.15 U/mgprot, and 16.98 ± 0.15 nmol/min/mgprot, which were significantly higher than those in the model group, while the TNF-α level was 102.18 ± 15.55 ng/L, which was significantly higher than that in the normal group (*p* < 0.05). The skin levels of these indices in mice in the VC, NMN, L and the NMN + L groups improved to varying degrees compared with the model group. Among them, the skin levels of the above indices in mice treated with NMN and LAB were the same as those in the normal group.

**TABLE 3:** Skin levels of T-SOD, CAT, IL-10, Na^+^-K^+^-ATPase, NAD^+^, and TNF-α in mice.

Group	T-SOD (U/mgprot)	CAT (U/mgprot)	Na^+^K^+^-ATP (U/mgprot)	NAD^+^ (nmol/min/mgprot)	TNF-α (ng/L)	IL-10 (pg/ml)
Normal	26.68 ± 6.52^a^	23.07 ± 3.41^a^	0.86 ± 0.15^a^	16.98 ± 2.49^a^	102.18 ± 15.55^b^	632.98 ± 82.99^a^
Model	7.21 ± 1.29^d^	15.70 ± 4.20^c^	0.22 ± 0.06^c^	13.35 ± 0.96^c^	154.01 ± 17.99^a^	105.40 ± 32.65^c^
VC	16.44 ± 3.79^c^	19.58 ± 2.90^b^	0.47 ± 0.15^b^	14.89 ± 1.58^bc^	145.79 ± 28.39^a^	146.99 ± 36.60^c^
NMN	18.62 ± 3.60^c^	21.48 ± 3.99^b^	0.60 ± 0.11^bc^	15.73 ± 0.84^ab^	115.35 ± 9.47^b^	259.06 ± 34.66^c^
L	18.68 ± 1.25^c^	21.52 ± 3.43^b^	0.62 ± 0.16^bc^	14.72 ± 0.42^bc^	117.23 ± 17.78^b^	223.79 ± 27.68^c^
NMN + L	25.83 ± 6.06^b^	23.80 ± 3.67^a^	0.75 ± 0.17^a^	16.53 ± 1.03^ab^	94.85 ± 14.57^b^	376.55 ± 63.75^b^

Values are mean ± standard deviation (*N* = 10/group). The difference in variance between the two groups was significant (*p* < 0.05).

^a-c^Mean values with different letters in the same column are significantly different (*p* < 0.05) according to Duncan’s honestly significantly different test. VC: mice treated with vitamin C (300 mg/kg).

NMN, mice treated with nicotinamide mononucleotide (300 mg/kg); L, mice treated with L. fermentum TKSN041 (1.0 × 10^9^ CFU/ml); NMN + L, mice treated with nicotinamide mononucleotide (300 mg/kg) and L. fermentum TKSN041 (1.0 × 10^9^ CFU/ml); T-SOD, total superoxide dismutase; CAT, catalase; TNF-α, tumor necrosis factor α; IL-10, interleukin 10.

### Skin and Liver mRNA and Protein Expression Levels of AMPK, NF-κBp65, IκB-α, SOD1, and CAT

**FIGURE 5:**
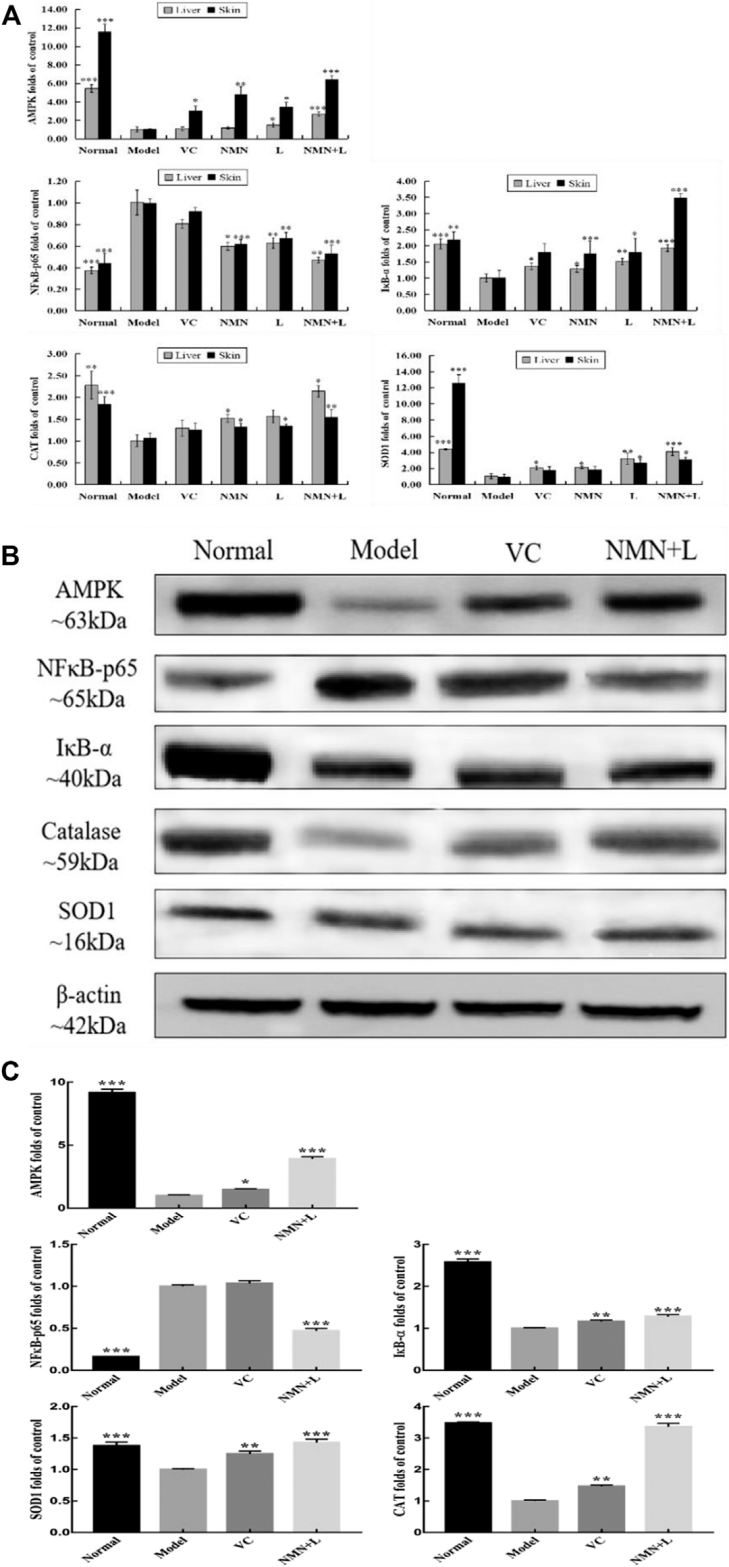
AMPK, NF-κBp65, IκB-α, SOD1, and CAT mRNA and protein expression levels in skin and liver tissues. **(A)** AMPK, NF-κBp65, IκB-α, SOD1, and CAT mRNA expression levels in skin and liver tissues; **(B)** protein stripe chart of AMPK, NF-κBp65, IκB-α, SOD1, and CAT in skin; **(C)** AMPK, NF-κBp65, IκB-α, SOD1, and CAT protein expression levels in skin. The data were calculated and analyzed using Pad Prism 7.0 (Graph Pad Software, La Jolla, CA, United States) software, group differences were also analyzed by one-way analysis of variance (ANOVA) followed by Duncan’s multiple comparison test. *p < 0.05 compared to the model group; **p < 0.01 compared to model group; ***p < 0.001 compared to the model group. VC: mice treated with vitamin C (300 mg/kg); NMN: mice treated with nicotinamide mononucleotide (300 mg/kg); L: mice treated with *L. fermentum TKSN041* (1.0 × 10^9^ CFU/ml); NMN + L: mice treated with nicotinamide mononucleotide (300 mg/kg) and *L. fermentum* TKSN041 (1.0 × 10^9^ CFU/ml).

AMPK, NF-κBp65, IκB-α, SOD1, and CAT are all biomarkers of oxidative stress and inflammatory (Yu et al., 2020). Real-time fluorescent quantitative PCR (RT-qPCR) and Western blotting are used to detect the mRNA expression and protein expression of the above genes. Figure 5A shows that UVB irradiation increased the mRNA expression levels of *NF-κBp65* in murine skin and liver and decreased the expression of *IκB-α*, AMP-activated protein kinase (*AMPK*), *SOD*, and *CAT* compared with those in the normal group. The mRNA expression levels of *IκB-α*, *AMPK*, *SOD*, and *CAT* in the skin and liver of the VC, NMN, L and NMN + L groups all increased to varying degrees compared with those in the model group, while the expression of *NF-κBp65* decreased. Figures 5B,C show that UVB irradiation increased the protein expression level of NF-κBp65 in murine skin and decreased the expression levels of IκB-α, AMPK, SOD, and CAT. However, the protein expression levels of NF-κBp65, IκB-α, AMPK, SOD, and CAT in the skin of the NMN + L group were significantly different from those in the model group. Among them, the mRNA and protein expression levels of the above indicators in the NMN + L group were close to those in the normal group.

### Skin and Liver mRNA Expression Levels of *PGC-1α*, *APPL1*, *mTOR*, *FOXO*, *TNF-α*, *IL-6*, *IL-10*, and *GSH*

**FIGURE 6:**
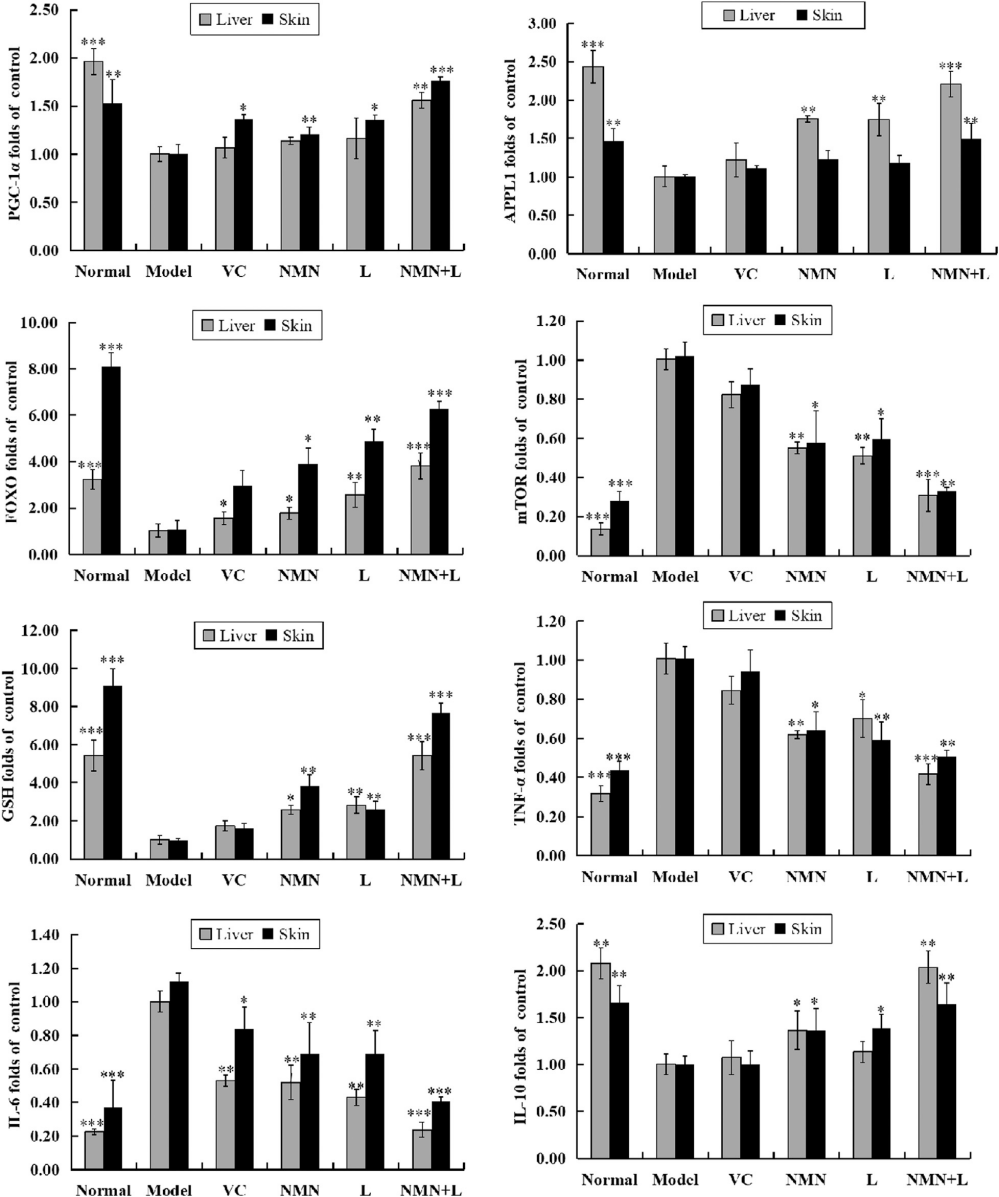
PGC-1α, APPL1, mTOR, FOXO, TNF-α, IL-6, IL-10, and GSH mRNA expression levels in skin and liver tissues. The data were calculated and analyzed using Pad Prism 7.0 (Graph Pad Software, La Jolla, CA, United States) software, group differences were also analyzed by one-way analysis of variance (ANOVA) followed by Duncan’s multiple comparison test. *p < 0.05 compared to the model group; **p < 0.01 compared to the model group; ***p < 0.001 compared to the model group. VC: mice treated with vitamin C (300 mg/kg); NMN: mice treated with nicotinamide mononucleotide (300 mg/kg); L: mice treated with *L. fermentum TKSN041* (1.0 × 10^9^ CFU/ml); NMN + L: mice treated with nicotinamide mononucleotide (300 mg/kg) and *L. fermentum TKSN041* (1.0 × 10^9^ CFU/ml).

In order to analyze the effects of ultraviolet radiation on oxidative stress and inflammation of the skin and liver more comprehensively, we also detected the skin and liver mRNA expression levels of PGC-1α, APPL1, mTOR, FOXO, TNF-α, IL-6, IL-10, and GSH by RT-qPCR. Figure 6 shows that the mRNA expression levels of PGC-1α, APPL1, FOXO, IL-10, and glutathione (GSH) were highest in the skin and liver of the normal group of mice, and the expression levels of mTOR, TNF-α, and IL-6 were the lowest. The expression levels of these indicators in the skin and liver of the model group showed a completely opposite trend compared with the normal group, and a significant difference was observed between the two. After treatment with VC, NMN, *L. fermentum* TKSN041 and NMN combined with *L. fermentum* TKSN041, the expression levels of PGC-1α, APPL1, FOXO, IL-10, and GSH increased in the skin and liver, while the expression levels of mTOR, TNF-α, and IL-6 decreased. The expression level of the NMN + L group was close to that of the normal group.

## Discussion

Exposure to UVB (290–320 nm) could result in histologic and clinical injuries such as skin aging, skin inflammation, infection and cancers (Wang et al., 2019). Some reports indicate that both NMN and lactic acid bacteria have a variety of biological activities and have a photoprotective effect on the skin damaged by UVB (Kim et al., 2019a; Zhao et al., 2020). However, there are few reports on exploring the improvement effect of the combination of these two substances on UVB-induced skin damage. In this regard, we assume that NMN combined with *L. fermentum* TKSN041 can also improve UVB-induced skin photodamage. For this reason, the mouse photodamage model is used to verify this conjecture.

Many researches have repoted that free radicals and oxidantive stress play an important pathophysiological role in the skin ageing. Therefore, the consumption of antioxidants becomes an important means of preventing or delaying the appearance of skin ageing (Lephart, 2016). Nicotinamide mononucleotide (NMN), a key NAD^+^ intermediate, has been shown to enhance NAD^+^ biosynthesis and ameliorate various pathologies in mouse disease models (Yoshino et al., 2011). In general, many lactic acid bacteria are belongs to probiotics, such as *Lactobacillus*, *Bifidobacterium*, *Streptococcus*, and *Enterococcus* genera. These probiotic strains usually possess some functional properties (antioxidant, assimilation of cholesterol, and alleviate diabetes, etc) (Papadimitriou et al., 2016). In our previous experiment, we found that the survival rates of *L. fermentum* TKSN041 in pH 3.0 simulated gastric juice and 0.3% bile salts were respectively 91.24 ± 1.12% and 15.81 ± 0.47%, showing good *in vitro* resistance, this result can indicate that *L. fermentum* TKSN041 have the ability to colonize the intestinal tract and have the potential to exert probiotic effects (Huang and Adams, 2004). In this study, the *in vitro* antioxidant evaluation method was used to evaluate the antioxidant capacity of NMN and *L. fermentum* TKSN041. The results show that the NMN combined with *L. fermentum* TKSN041 had higher free radical scavenging abilities than NMN or *L. fermentum* TKSN041 alone. Among them, with the continuous research on the gut-skin axis, the research on the protective effect of intracellular products of lactic acid bacteria on skin is also increasing. Application of the lysates of *Lactobacillus rhamnosus* could increase the expression of tight junction proteins, and then improve skin barrier function (Jung et al., 2019). The protective effect of intracellular products of lactic acid bacteria on skin may be related to beneficial intracellular metabolites, such as the protective effect of short chain fatty acids (SCFAs) on inflammatory diseases including arthritis and allergy (Kim et al., 2014b), but its specific mechanism needs to be further studied and determined. Based on the above research, it is of great significance to explore the beneficial effects of *L. fermentum* TKSN041 combined with NMN on the skin through subsequent *in vivo* experiments.

The weight of tissues and organs, particularly changes in the weight of vital organs, such as the liver, brain, and spleen, is an important indicator of animal aging. Aging usually causes weight loss in most organs, which affects the body’s immune response and metabolic activities (Corsetti et al., 2018). The liver is one of the most sensitive organs during the aging process. The murine organ indices and the pathological morphology of the liver directly reflect the structural changes and functions of the organs and are significant for evaluating the functional characteristics of tested samples (Liu et al., 2012; Bhatia et al., 2014). In this study, the liver organ indices and the hepatic pathological morphology in the model group of mice decreased significantly after UVB irradiation, indicating that UVB irradiation not only directly accelerated skin aging but also indirectly caused liver aging, which may be related to oxidative stress or inflammation induced by UVB (Coltart et al., 2013). However, after intragastric administration of NMN combined with *L. fermentum* TKSN041, the liver organ indices and the hepatic pathological morphology of the mice improved significantly, indicating that NMN combined with *L. fermentum* TKSN041 maintained the normal weight of the murine liver and delayed liver aging. Previous studies on liver function of NMN or *L. plantarum* AR501 have also obtained the same result, that is, these two substances can reduce the oxidative stress and inflammatory damage of the liver (Lin et al., 2018; Assiri et al., 2019).

Histopathological observations can quickly determine serious skin damage caused by UV rays. H&E, Masson staining, and toluidine blue staining are often used to observe pathological changes in skin. A recent study have verified that UVB irradiation caused the skin dermis became thinner after H&E staining (Blackstone et al., 2020), our study found the same result. In our study, the number of collagen fibers in the skin of mice with UVB-induced skin damage decreased, and at the same time, collagen fibers appeared atrophy, breakage and stickiness (Feng et al., 2019). Otherwise, the number of mast cells in the dermis layer increases significantly after UVB irradiation, which indicate the aggravation of skin inflammation (Kim et al., 2014a). Interestingly, the pathological morphology of the skin improved considerably after treatment with NMN combined with *L. fermentum* TKSN041.

The oxidative stress response is an important factor in UVB-induced skin aging (Cavinato and Jansen-Dürr, 2017). Under normal circumstances, the generation and removal of oxygen free radicals are in a balanced state. When stimulated by exogenous sources, the body generates a large amount of oxygen free radicals due to local hypoxia, resulting in cell apoptosis and damage (Di Meo and Venditti, 2020). SOD and CAT are important free radical scavenging enzyme, some reports have found that oxidative stress caused by ultraviolet radiation will rapidly reduce the activity of these enzymes (Oliveira et al., 2019). Our experiments also found that the SOD and CAT enzyme activities in mice after UVB radiation significantly decreased. We also found that UVB irradiation greatly reduces the level of GSH, and a large reduction in GSH will aggravate the skin damage caused by the active oxygen generated by UVB (Cai et al., 2019). MDA is the final product of lipid oxidation, its content reflects lipid peroxidation and indirectly indicates the degree of cell damage. AGEs are the end products of nonenzymatic glycosylation reactions. Senile diseases are closely related to modifications in AGE proteins (Zimmerman et al., 2017; Song et al., 2018), Our current results found that the content of MDA and AGEs in serum and skin of UVB induced skin injury mice significantly increased, indicating that UVB irradiation accelerated lipid peroxidation and glycosylation reactions. However, NMN combined with *L. fermentum* TKSN041 increased T-SOD and CAT activities in murine serum and skin tissue, increased SOD and CAT mRNA and protein expression levels in liver and skin, and reduced the content of AGEs and MDA in serum. These results show that NMN combined with LAB resists the oxidative stress of the skin caused by UVB irradiation in mice by increasing the activities of antioxidant enzymes and improving the overall antioxidant level of the body.

It has been reported that a key mechanism of the anti-aging effect of NMN is to reverse the decline of age-related mitochondrial function. NAD^+^, as a rate-limiting substrate for the sirtuin enzyme, is a key regulator of the pro-survival pathway and mitochondrial function in endothelial cells. Evidence indicates that the availability of intracellular NAD^+^ decreases with age or exposure to UVB irradiation, thereby promoting aging of the skin or body. Supporting this theory, enhancing NAD^+^ biosynthesis extends the healthy life span of mice and can reverse a variety of age-related organ dysfunctions in elderly mice (Bonkowski and Sinclair, 2016; Rajman et al., 2018). As a coenzyme, Na^+^-K^+^-ATPase has the effect of improving the body’s metabolism and participating in fat, protein, sugar, nucleic acid, and nucleotide metabolism. It is also the main source of energy in the body, providing the energy for absorption, secretion, muscle contraction, and biochemical synthesis. Studies have shown that NMN restores NAD^+^ and ATP levels, reduces oxidative stress in blood vessels, maintains the antioxidant system of GSH and thioredoxin, inhibits apoptosis, and improves energy metabolism disorders induced by mitochondrial inhibitors (Wang et al., 2020). In this study, the levels of NAD^+^ and Na^+^-K^+^-ATPase in the skin tissue of the model group were significantly lower than those in the normal group, indicating that UVB irradiation causes skin energy metabolism disorders (An et al., 2012; Hong et al., 2020). After treatment with NMN combined with *L. fermentum* TKSN041, the levels of NAD^+^ and Na^+^-K^+^-ATPase in the skin increased significantly, indicating that oral administration of NMN combined with *L. fermentum* TKSN041 maintained a balanced energy metabolism in mice, thus reducing oxidative damage. Studies have shown that mammals can convert NMN into NAD^+^, and the mechanism may be related to deamidation of bacterial activation (Shats et al., 2020), this research may indicate that the *L. fermentum* TKSN041 in our study played a role in improving skin injury by promoting the synthesis of NMN into NAD^+^.

The skin produces a strong oxidative stress response when exposed continuously to UV light by releasing a large amount of reactive oxygen species (ROS). ROS act as an upstream signal to activate the NF-κB-mediated inflammatory pathway, thereby making the skin appear dry with itching, erythema, edema, and other inflammatory symptoms (Forrester et al., 2018). Under normal circumstances, the nuclear transcription factor NF-κB and its inhibitory protein IκB combine and are stored in the cell during rest. Once NF-κB is activated, it is transferred from the cytoplasm to the nucleus, thereby further increasing release of the pro-inflammatory cytokines TNF-α, IL-6, IL-12, cyclooxygenase-2, and inducible nitric oxide synthase, which induce inflammatory damage (Neacsu et al., 2015). Various studies have already demonstrated that lactic acid bacteria (Galli et al., 2018) as well as NMN (Fan et al., 2011) distinctly interrupts the nuclear translocation of NF-κB by inhibiting the activation of IKK kinase which results in the down-regulated phosphorylation of IκB, which leads to the abolishment of the separation of NF-κB from I-κB. Similar to these studies, NMN combined with *L. fermentum* TKSN041 upregulated the IκB-α mRNA and protein expression in the skin and liver tissues, thereby inhibiting activation of the NF-κBp65 signaling pathway.

To investigate the degree of skin inflammation induced by UVB irradiation, we measured the levels of TNF-α and IL-6 associated with activating NF-κB. The results demonstrated that NMN combined with *L. fermentum* TKSN041 downregulated the expression levels of the pro-inflammatory cytokines TNF-α and IL-6 from serum, skin tissue, and mRNA, and upregulated the expression of the inflammatory cytokine IL-10. Both TNF-α and IL-6 are important pro-inflammatory cytokine (McHale et al., 2018; Zhang et al., 2018), and IL-10 is an important negative regulatory cytokine that blocks multiple links in immune inflammatory reactions (Vanderwall et al., 2018). On the one hand, previous studies found that UV radiation can cause the secretion and expression of TNF-α and IL-6, but reduce the secretion and expression of IL-10 (Lee et al., 2018). On the other hand, some researches verified lactic acid becteria or NMN can reduce the levels of TNF-α, IL-6 and increased IL-10 level (Sims et al., 2018; Li et al., 2020), which may provide a basis for our current experimental results.

AMPK is a serine/threonine protein kinase and a heterotrimer comprised of α and β catalytic subunits and regulatory subunit γ. It is mainly involved in regulating sugar, lipid, and energy metabolism, and studies have shown that activating AMPK inhibits inflammation and oxidative stress (Salt and Hardie, 2017). In addition to maintaining cellular energy homeostasis, experiments have shown that inhibiting AMPK activity significantly increases the expression levels of the inflammatory factors TNF-α, IL-1β, and IL-6, thereby increasing inflammation damage (Gurung et al., 2020). In addition, high AMPK expression levels in young cells promote the activities of factors, such as SIRT1, FOXO, and PGC-1α, thereby inhibiting NF-κB activity. After cells age, NF-κB signaling is enhanced due to decreased AMPK activity (Li et al., 2015). In this experiment, intragastric treatment of NMN combined with *L. fermentum* TKSN041 significantly increased AMPK mRNA and protein expression levels in the liver and skin tissues, indicating that NMN combined with *L. fermentum* TKSN041 effectively promoted cellular energy synthesis and reduced oxidative stress. AMPK relies on the metabolites and common substrates associated with ATP metabolism, while NMN accelerates ATP production by promoting NAD^+^ synthesis (Hsu and Burkholder, 2016). On the other hand, studies have verified that *Lactobacillus* can positively regulate the intestinal microbiota, reduce the number of gram-negative bacteria, and increase the level of short-chain fatty acids, and they also have the ability to activate the AMPK pathway in mammalian cell cultures via phosphorylation (Lew et al., 2020; Li et al., 2021). Although there are many studies on lactic acid bacteria and NMN that activate AMPK signaling pathways alone, there are few studies on the combination of these two substances to activate AMPK. However, the results of this study suggest that these two substances can synergistically promote the activation of AMPK, but further experiments are needed to explore its specific mechanism.

As an upstream gene of AMPK, APPL1 regulates the inflammatory response of cells, antioxidation, and arteriosclerosis (Zhou et al., 2009). Here we show that, after treatment of NMN combined with *L. fermentum* TKSN041, the APPL1 level increased with the activation of AMPK. UVB is reported to activate mTOR signaling, it plays a role in the development of skin cancer, but activated AMPK can inhibit mTOR activity (Carr et al., 2012; Sanli et al., 2014). Our results also confirmed that UVB irradiation downregulated the expression of AMPK and then decreased the expression of mTOR. However, intragastric administration of NMN combined with *L. fermentum* TKSN041 reversed this change, which may be related to the regulation effects of mTOR signaling pathway by lactic acid bacteria (Fu et al., 2017).

Finally, we found UVB irradiation reduced the FOXO, and PGC-1α mRNA expression levels in murine skin and liver. However, the mRNA expression levels of FOXO, and PGC-1α in the skin and liver of mice increased significantly after intragastric administration of NMN combined with *L. fermentum* TKSN041. FOXO is associated with cell death and oxidative stress (Lee and Dong, 2017). PGC-1α is a regulator of AMPK that participates in mitochondrial biosynthesis by regulating the body’s adaptive heat production, glucose and lipid metabolism, and blood sugar balance; it improves mitochondrial respiration and regulates fatty acid oxidation (Zhao et al., 2014). It has been reported that *Lactobacillus paracasei* can promote lipid oxidation by metabolizing acetyl-CoA and AMP, and then upregulating the AMPK/PGC-1α/PPARα pathway (Araújo et al., 2020), this can provide some reference for our current results.

## Conclusion

In summary, NMN combined with *L. fermentum* TKSN041 had good *in vitro* antioxidant capacity and improved UVB-induced skin damage in mice. The possible mechanism may be that the combination of NMN and *L. fermentum* TKSN041 that activated the AMPK signaling pathway, thereby inhibiting activation of the NF-κB signaling pathway and reducing the damage caused by inflammatory mediators to mice. In addition, activated AMPK reduced oxidative damage of the skin and improved the body’s overall antioxidant capacity by regulating the levels of relevant oxidative stress indicators in the blood, liver, and skin. This study is an important reference for preventing and treating skin damage caused by UVB and provides a theoretical basis and source of available strains for the development of health foods that combine NMN and LAB.

## Data Availability

The original contributions presented in the study are included in the article/Supplementary Material, further inquiries can be directed to the corresponding authors.
